# Review: apoptotic mechanisms in bacterial infections of the central nervous system

**DOI:** 10.3389/fimmu.2012.00306

**Published:** 2012-10-04

**Authors:** Geetha Parthasarathy, Mario T. Philipp

**Affiliations:** Division of Bacteriology and Parasitology, Tulane National Primate Research Center, Tulane UniversityCovington, LA, USA

**Keywords:** apoptosis, CNS, bacterial infections, inflammation, meningitis

## Abstract

In this article we review the apoptotic mechanisms most frequently encountered in bacterial infections of the central nervous system (CNS). We focus specifically on apoptosis of neural cells (neurons and glia), and provide first an overview of the phenomenon of apoptosis itself and its extrinsic and intrinsic pathways. We then describe apoptosis in the context of infectious diseases and inflammation caused by bacteria, and review its role in the pathogenesis of the most relevant bacterial infections of the CNS.

## Introduction

In this article we review the apoptotic mechanisms most frequently encountered in bacterial infections of the central nervous system (CNS). We focus specifically on apoptosis of neural cells (neurons and glia), and provide first an overview of the phenomenon of apoptosis itself and its extrinsic and intrinsic pathways. We then describe apoptosis in the context of infectious diseases and inflammations caused by bacteria, and review its role in the pathogenesis of the most relevant bacterial infections of the CNS.

## Apoptosis and other cell death mechanisms: overview

Apoptosis, a type of programmed cell death, is a highly controlled physiological process that plays an essential role in both pathological and normal conditions in multicellular organisms. The term was coined in 1972 by Kerr, Wyllie, and Currie to designate a process that is morphologically distinct from cell death due to necrosis—a phenomenon that was well described at the time (Kerr et al., 1972). Cells undergoing apoptosis typically display shrinkage, membrane blebbing, nuclear condensation, and DNA cleavage resulting in cellular fragmentation, with the cytoplasm retained in subcellular apoptotic bodies. These changes are accompanied by translocation of the phospholipid phosphatidylserine from the inner to the outer surface of the cell membranes (Dockrell, 2001). The process is confined to either single cells or a cell cluster, and is energy driven, gene regulated, and controlled (Elmore, 2007). Necrosis, on the other hand, results in cell swelling, mitochondrial rupture, loss of membrane integrity, and subsequent release of cell contents into the surroundings. It often occurs in contiguous cells, and unlike apoptosis, it is a passive process and is not energy driven. It should be noted that apoptotic cells are normally eliminated by phagocytic cells. However, in the absence of scavengers to clean up apoptotic cells, transition to a secondary or late stage necrosis occurs (Elmore, 2007; Silva, 2010). In addition to apoptosis and necrosis, there are other cell-death processes such as autophagy, programmed necrosis, etc. Autophagy, literally meaning “eating oneself” (Kelekar, 2005) is a non-apoptotic programmed process that often occurs in cells that undergo nutrient deprivation or cellular insults, resulting in formation of a double membrane around the damaged organelle to be eliminated. This vesicle, termed autophagosome eventually fuses with the cellular lysosome to form an autolysosome (or autophagolysosome), culminating in elimination of the contents within. There is still debate on whether autophagy is primarily a survival or a death pathway, as evidence supporting both roles has been obtained (Edinger and Thompson, 2004; Kelekar, 2005). Programmed necrosis, on the other hand, is described as being solely a death pathway, and contrary to the “accidental” primary necrosis described previously, in this process cell death occurs, as the name implies, in a programmed fashion. One such type of regulated necrosis, termed necroptosis is the most widely studied. In necroptosis, signals that lead otherwise to apoptosis, in this instance end in cell death but with the cells acquiring a necrotic morphology, and without caspase activation (described below) (Galluzzi and Kroemer, 2008; Vandenabeele et al., 2010; Han et al., 2011). It is to be noted that endpoints of primary necrosis, secondary necrosis (occurring after apoptosis), or programmed necrosis are morphologically impossible to distinguish, although kinetics may vary (Vanden Berghe et al., 2010). Detailed reviews of these processes are described in the indicated references and in others (Boujrad et al., 2007).

Apoptosis occurs during normal embryonic development, organ metamorphosis, cell turnover, and aging, and is required to maintain tissue homeostasis (Elmore, 2007). For instance, during neural development, neurons are overproduced in large number, to then undergo programmed cell death in approximately 50% of the population to ensure optimum synaptic connections, pattern formation, and removal of excessive neurons (Burek and Oppenheim, 1996; Nijhawan et al., 2000). In amphibians, during the transition from larva to adult, apoptosis of larval tissue cells proceeds along with the production of adult organs (Ishizuya-Oka, 2011). In the immune system, apoptosis plays a vital role in induction of T-lymphocyte tolerance during development, and in lymphoproliferation control of activated lymphocytes (Dockrell, 2001). Additionally, damaged cells undergo apoptosis to be replaced by cell proliferation leading to tissue homeostasis (Warner, 1997). The stimuli that initiate apoptosis are varied—including exposure to proteins (hormones) or chemicals, e.g., chemotherapy drugs, energy sources such as radiation, and many others. However, unlike the numerous stimuli for apoptosis, the process of apoptosis itself proceeds largely following two pathways—the extrinsic and the intrinsic pathways.

### The extrinsic pathway

In this process, specific receptors on the cell surface interact with ligands to initiate apoptosis. Well known examples include the tumor necrosis factor receptor-1(TNFR1) that binds TNF and the Fas receptor (FasR) that binds to Fas ligand (FasL). Others include the death receptors (DR) 3, 4, and 5 that bind Apo3L (DR3) or Apo2L (DR4/5) (Ashkenazi and Dixit, 1998; Gao and Kwaik, 2000). Ligand binding leads to clustering of receptors and recruitment of adapter molecules like Fas-associated death domain (FADD) or TNF receptor-associated death domain (TRADD) to the cytoplasmic domain of the receptor, leading to subsequent recruitment and activation of caspases (caspase-8 in this instance)^1^.

Caspases (cysteine-dependent aspartic acid specific proteases) are central to the process of apoptosis. They are broadly classified into initiators (caspase-2, 8, 9, 10) or effectors (caspase-3, 6, 7) although others such as inflammatory caspases (1, 4, and 5) process pro-inflammatory cytokines. Briefly, activation of an initiator caspase leads to activation of effector caspases that in turn activate molecules like caspase-activated DNase (CAD) or inactivate poly ADP ribose polymerase (PARP—a DNA repair enzyme), leading to breakdown of cytokeratin, cytoskeleton, nuclear proteins, and DNA, resulting in cell death. Although apoptosis is generally considered irreversible, there are instances where, when the stimulus is too weak or is removed in a timely fashion, the process may be reversed (Geske et al., 2001; Tang et al., 2012). However, while this is a possibility, it is not clear how often this occurs *in vivo*.

### The intrinsic pathway

The intrinsic pathway is initiated through mitochondria-associated alterations. Apoptotic stimuli that activate this process result in change of mitochondrial membrane permeability, with the release of several proteins such as cytochrome c and smac/DIABLO (second mitochondria-derived activator of caspase/direct inhibitor of apoptosis-binding protein with low pI) into the cytosol. Cytochrome c binds apoptotic protease activating factor (Apaf-1) and pro-caspase-9 forming the apoptosome complex, while smac/DIABLO bind and inhibit inhibitors of apoptosis proteins (IAPs). Activation of caspase-9 leads to activation of the downstream effector caspases, DNA fragmentation and death. Another pro-apoptotic protein that is released from the mitochondria later during the apoptotic process is the apoptosis-inducing factor (AIF). Like CAD it translocates to the nucleus to cause DNA damage but unlike CAD, it is not caspase-dependent. Therefore, AIF release is often considered as a hallmark of caspase-independent programmed cell death.

The above-described mitochondria-associated events during the apoptotic process are regulated by the Bcl-2 (B-cell lymphoma 2) family of proteins, with members that are either pro- or anti-apoptotic. The pro-apoptotic protein Bad (Bcl-2-associated death promoter) in a non-phosphorylated state binds and sequesters the anti-apoptotic Bcl-2. When Bad is phosphorylated, however, it is sequestered within the cytosol while the free Bcl-2 inhibits the release of cytochrome c (Shou et al., 2004). Other members that might play similar (but not necessarily identical) roles in stabilization/destabilization of mitochondrial membrane permeability include the pro-apoptotic Bcl-10, Bax (Bcl-2-associated X protein**)**, and Bak and the anti-apoptotic Bcl-x, as well as others. A complete account of such events is described extensively elsewhere (Elmore, 2007; Kaufmann et al., 2012).

While the extrinsic and intrinsic pathways seem distinct in their initiators and effectors, this is not always so. In certain cell types, the Fas-mediated signal requires amplification of the caspase cascade to cause death, and this is achieved by caspase-8 activation of the pro-apoptotic Bid (BH3-interacting domain death agonist), which leads to increase in the mitochondrial membrane permeability and the associated cascade of events (Kaufmann et al., 2012).

Another molecule that plays a role in both pathways is the tumor suppressor p53. p53, which is encoded by the *TP53* gene, is a protein with very complex functions. It is required for normal genomic maintenance and plays an important role in cell-cycle arrest, DNA repair, senescence, angiogenesis, cell differentiation, metabolism, autophagy, and apoptosis (Amaral et al., 2009; Vousden, 2009; Vousden and Ryan, 2009). p53 levels are kept low in normal healthy cells, but in cells undergoing stress its level can be upregulated, predominantly through non-transcriptional mechanisms like stabilization (Amaral et al., 2009; Takahashi et al., 2011). In the extrinsic apoptotic pathway, p53 can activate expression of the *FAS* gene (Bouvard et al., 2000) as well as influence the surface expression of its product by trafficking Fas from the Golgi complex (Bennett et al., 1998). The gene that encodes the DR5, a receptor that binds TRAIL/Apo2L, is also a p53-regulated gene (Wu et al., 1997). In the mitochondria-related intrinsic pathway, several of the Bcl-2 family of proteins (both pro- and anti-apoptotic) is regulated by p53-mediated transcription. Cytosolic p53, in non-transcriptional mediated mechanisms, also influences the levels of the Bcl-2 family of proteins in the mitochondria through binding, leading to either repression or activation (reviewed in Speidel, 2010).

Apoptosis is a complex process that is triggered by numerous and diverse stimuli and is effected by several mediators in various cells and tissues. Thus, it is imperative that apoptotic mechanisms are delineated in particular settings, as one type does not fit all scenarios.

## Role in disease

Apoptosis, like any other biological mechanism, is subject to errors and dysregulation. Dysregulated apoptosis, either too much or too little, can lead to disease and pathological conditions. Mutations in the p53 gene have been seen in at least 50% of tumors. Low rate of apoptosis can lead to survival of abnormal cells, eventually leading to tumor formation, autoimmune diseases and others. Examples include Leukemia, Graves' disease, and rheumatoid arthritis. On the other hand, excessive, unregulated apoptosis leads to many neurodegenerative diseases, multiple sclerosis, Type I diabetes, myelodysplastic syndromes, etc. (Peter et al., 1997). While these diseases and conditions are caused by inborn errors, environmental, and unknown triggers, pathogens and infections can also cause apoptosis of eukaryotic cells leading to excessive—apoptosis conditions, as the affected cells normally would not undergo cell death if left undisturbed. A well-known example is human immunodeficiency virus (HIV), which causes apoptosis of CD4 lymphocytes. Many other viruses and bacteria similarly induce apoptosis of different eukaryotic cells and tissues, leading to syndromes and chronic conditions. In this review, we place emphasis on CNS infections by bacteria and provide a compilation of the apoptotic mechanisms these bacteria induce.

## Bacterial CNS infections and apoptosis

CNS infections are among the most morbid forms of infectious disease both because of their high associated mortality and their neurological complications with ensuing long-term sequelae in the survivors. CNS infections can be broadly classified as causing meningitis (inflammation of the meninges), encephalitis or myelitis (inflammation of the brain or spinal cord, respectively), and brain abscesses. In the United States alone, between the years 1988 and 1997, 19,000 encephalitis cases were reported annually with 1400 deaths, and a healthcare cost of $650 million/year. (Khetsuriani et al., 2002). In the year 2006, this increased to about $1.2 billion with about 72,000 meningitis-related hospital admissions (Holmquist et al., 2006). Occurrence of brain abscesses on the other hand is comparatively sparse and accounts for 2000 cases annually, although the associated mortality rate is around 20% (Yang, 1981; Calfee and Wispelwey, 2000; Carpenter et al., 2007; Somand and Meurer, 2009).

The microorganisms that cause CNS infections are mainly bacteria, viruses, protozoa, and fungi. Although viral meningitis is more common than that caused by bacteria the latter is more deadly. In the 2006 study mentioned previously (Holmquist et al., 2006), bacterial meningitis accounted for 21.8% of the cases with a mortality rate of 8% whereas viral meningitis originated 54.6% of the hospitalizations but with a drastically lower mortality rate of 0.6%. Observational studies in humans over a long period of time have shown several long-term sequelae associated with CNS infections in the survivors. Cognitive impairment, hearing loss, motor neuron deficits, sleeping disorders, and seizures have all been reported (Schmidt et al., 2006a; Chuang et al., 2010; Mook-Kanamori et al., 2011). Other complications include tiredness/fatigue, headache, and psychiatric disturbances like depression, anxiety, mood swings, and others (Fallon et al., 1998; Pontrelli et al., 1999; Schmidt et al., 2006b). Added to this is the financial impact on the individual, family, and the health care system, making these infections a serious societal problem.

The bacterial species that cause CNS infections are varied and numerous. *Streptococcus pneumoniae* (pneumococcus), *Hemophilus influenzae* B, and *Neisseria meningitidis* (meningococcus) are among the most common agents of bacterial meningitis in the US, along with Group B *Streptococcus* and *Listeria monocytogenes* (Schuchat et al., 1997). *Mycobacterium tuberculosis*, *Treponema pallidum* (the agent of syphilis), the Lyme disease spirochete *Borrelia burgdorferi*, *Rickettsiae* and others may cause meningitis and/or encephalitis, while *Staphylococcus aureus* and *Streptococcus* species are common causative agents of brain abscesses (Garg et al., 2009; Bartzatt, 2011; Beckham and Tyler, 2012). Evidence from several experimental studies has demonstrated loss of brain cells following infection with these agents. *S. pneumoniae* infection leads to apoptosis of hippocampal and cerebellar neurons, brain microvascular endothelial cells, and microglia (Braun et al., 2002; Bermpohl et al., 2005). *B. burgdorferi* infection in experimental neuroborreliosis results in loss of oligodendrocytes and neurons in the nonhuman primate brain, while *Brucella abortus* has been shown to induce astrocyte gliosis and apoptosis (Ramesh et al., 2008; Garcia Samartino et al., 2010). Loss of oligodendrocytes may lead to axonal degeneration of associated neurons (Edgar and Nave, 2009), while astrocyte loss may result in impaired metabolite transport across the blood-brain barrier, in a manner that affects efficient neuronal function. Loss and functional impairment of neural (neuronal and glial) cells may cause the long-term sequelae of bacterial CNS infection, even after removal of the causative agent with antibiotics. Although neuroregeneration in adult brain is known to occur following injury, studies indicate that it may be insufficient and not fully replenish the cell numbers required for complete restoration of function (Nakaguchi et al., 2011). In addition, infection may also lead to apoptosis of neural stem and progenitor cells, thus impairing neurogenesis (Hofer et al., 2011). Other than in neural cells, apoptosis can also occur in the cells of the immune system that are recruited during infection, resulting mostly in a beneficial effect to the host by limiting inflammation and injury (Nau et al., 1998). Thus, it is important that mechanisms mediating such cell loss be understood for effective interventions and better therapeutics. Here we evaluate the current knowledge of the apoptotic mechanisms elicited in neural cells and brain endothelial cells by CNS invading bacteria. Excluded from our analysis are bacteria that may cause CNS disease but for which neural or brain endothelial cell apoptosis has not been described.

### *streptococcus pneumoniae* (SP)

*S. pneumoniae*, a gram-positive, catalase-negative bacterium is one of the most common causes of bacterial meningitis in the US and Europe (Table 1). Infection with this bacterium can result in high incidence of severe neurological sequelae and large fatality rates (Schuchat et al., 1997; Braun et al., 1999; Mook-Kanamori et al., 2011). Autopsies of patients with fatal bacterial meningitis have shown that the majority of such patients (26/37) had apoptotic neurons in the dentate gyrus region of the brain (Nau et al., 1999). This apoptotic phenomenon has been replicated in several animal models of SP meningitis (Zysk et al., 1996; Braun et al., 1999; Bifrare et al., 2003; Hoffmann et al., 2011) and is a hallmark of SP-mediated CNS infection. Considerable attention has been paid over the years to the elucidation of neuronal and non-neuronal apoptotic mechanisms due to SP infections and the results show that a myriad of factors is involved. Inflammation, a commonly seen phenomenon in most CNS infections, has been shown to play a role in SP-mediated neuronal apoptosis. Anti-inflammatory therapy by administration of anti-CD18 antibody (a neutrophil marker) reduced the number of apoptotic neurons in a rabbit model of SP meningitis (Zysk et al., 1996; Braun et al., 1999). As neutrophils are a source of oxidants and other inflammatory mediators, reduction in their number in the cerebrospinal fluid (CSF) presumably induced a neuro-protective response by down-regulating inflammation. This was confirmed when CSF obtained from infected hosts and depleted of bacteria and leukocytes, but containing an inflammatory milieu, was able to induce apoptosis when administered *in vitro* to human cortical neurons (Braun et al., 1999). A role for caspases in neuronal apoptosis was implicated by the up-regulation of activated caspase-3 in tissue sections of dentate gyrus neurons in SP-infected rabbits. Also, *in vitro* neuronal apoptosis induced by CSF from infected hosts was mitigated in the presence of the caspase inhibitor z-VAD-fmk thus implying that inflammatory mediators released during SP infection were able to induce caspase activation and neuronal death (Braun et al., 1999). A similar result was obtained in an infant rat model of SP meningitis, where an up-regulation of activated caspase-3 was seen in immature dentate gyrus neurons, and neuronal apoptosis decreased by about 50% with a selective caspase-3 inhibitor (Gianinazzi et al., 2003). However, SP meningitis-mediated apoptosis in CNS has been shown to induce caspase-independent pathways as well and will be described shortly.

**Table 1:** **CNS apoptotic mechanisms in bacterial infections**.

**Bacteria**	**Gram stain**	**Cell apoptosis**	**Bacterial factors**	**Host factors**	**References**
*Streptococcus pneumoniae*	Gram positive	Neurons, microglia, endothelial cells	Hydrogen peroxide, pneumolysin, pneumococcal cell wall	Inflammatory mediators, caspase dependent and independent pathways, TLR2-dependent and independent mechanisms, thrombopoietin, phosphatidyl choline biosynthesis, iNOS, excitatory amino acids?	Zysk et al., 1996; Braun et al., 1999, 2001, 2002, 2007; Tumani et al., 2000; Winkler et al., 2001; Gianinazzi et al., 2003; Kolarova et al., 2003; Mitchell et al., 2004; Zweigner et al., 2004; Bermpohl et al., 2005; Hoffmann et al., 2006, 2011; Braun, 2009
Group B *Streptococcus*	Gram positive	Neurons, microglia	Secreted protein factor, non-heat labile factors, beta-hemolysin	Reactive oxygen species, NO, TNF, TLR2, excitatory amino acids, caspase-dependent and independent pathways	Leib et al., 1996a,b; Bogdan et al., 1997; Lehnardt et al., 2006, 2007; Reiss et al., 2011
*Brucella abortus*	Gram negative	Astrocytes	L-Omp19	TNF/TNFR1-mediated extrinsic pathway	Garcia Samartino et al., 2010
*Listeria monocytogenes*	Gram positive	Neurons	Listeriolysin		Schluter et al., 1998; Parra et al., 2008
*Staphylococcus aureus*	Gram positive	Neurons	Lipoteichoic acid, muramyl peptide?	Glial ROS, RNS via TLR2; neuronal caspase pathways (3 and 8)	Kinsner et al., 2005, 2006
*Borrelia burgdorferi*	Gram negative	Neurons, oligodendrocytes, astrocytes	Lipoproteins?	Inflammatory pathway in oligodendrocyte apoptosis, microglial factors in neuronal apoptosis (and neuronal p53?)	Ramesh et al., 2003, 2012; Bernardino et al., 2008; Myers et al., 2009
*Chlamydophila pneumoniae*	Gram negative	Neuronal cells	–	Microglial TNF and IL-6	Boelen et al., 2007a,b
*Neisseria meningitidis*	Gram negative	Brain microvascular endothelial cells	–	Caspase-dependent pathway (caspase-3)	Constantin et al., 2002; Schubert-Unkmeir et al., 2007
*Escherichia coli* K1	Gram negative	Neurons, endothelial cells	Hcp1	Caspase-8; NO	Spreer et al., 2006; Mittal et al., 2010; Zhou et al., 2012
*Klebsiella pneumoniae*	Gram negative	Neurons	–	Microglial production of TNF and IL-1β	Wen et al., 2007; Chiu et al., 2011; Wu et al., 2011
*Rickettsia rickettsii*	Gram negative	Cerebellar granule, neurons	–	–	Joshi and Kovacs, 2007

SP contains several virulence factors—all playing an important role in pathogenesis. Hydrogen peroxide, the cytolytic toxin pneumolysin, and the gram-positive cell wall, composed of peptidoglycan, teichoic, and lipoteichoic acid (LTA), are well-documented virulence factors in SP infections. Direct incubation of bacteria/bacterial factors with brain cells has shown that SP can induce divergent pathways of apoptotic cell death through different virulence factors. As with whole bacteria, pneumolysin and hydrogen peroxide both influence intracellular calcium release and the release of AIF from mitochondria, leading to caspase-independent cell death in both microglia and neurons (Braun et al., 2001, 2002). On the other hand, pneumococcal cell wall (PCW), a major pro-inflammatory compound (Tuomanen et al., 1985) causes apoptosis through the classical caspase-dependent pathway (Mitchell et al., 2004). In addition to inducing caspase-independent pathways of apoptotic cell death, pneumolysin was shown to actively suppress caspase-dependent pathways by the upregulation of X-chromosome linked inhibitor of apoptosis protein (XIAP), a caspase inhibitor (Braun et al., 2007). A similar dual mechanism also exists in apoptosis of brain microvascular endothelial cells (BMECs) by SP (Bermpohl et al., 2005). Cell death by various pathways in response to SP infection is time-dependent. Pneumococci, pneumolysin, and hydrogen peroxide induce caspase-independent apoptosis early in the infection while PCW induces the classical caspase-dependent apoptotic pathway much later (Mitchell et al., 2004). This ability of PCW to effect apoptosis has implications in post-treatment complications, as lytic antibiotics can lead to increased release of bacterial cell wall, resulting in further inflammation and injury.

While the role of SP bacterial factors in inducing CNS apoptosis is well known, the identification of host receptors in eukaryotic brain cells is beginning to be addressed. PCW-induced apoptosis requires host TLR2, as TLR2-deficient mice display reduced apoptosis of primary BMECs (Bermpohl et al., 2005). In contrast, pneumolysin-induced neuronal apoptosis was shown to be independent of TLR4, and BMEC apoptosis induced by this same virulence factor involved neither TLR2 nor TLR4 (Bermpohl et al., 2005; Braun et al., 2007).

Additional host components that might play a role in SP-induced apoptosis are phosphatidylcholine (PC) and thrombopoeitin. In human microglia, neurons, and brain endothelial cells, SP infection was shown to inhibit PC (a component of the cell membrane) synthesis; inhibition of PC synthesis is a known apoptotic process (Ramos et al., 2000). PC is synthesized in three key enzymatic steps, involving choline kinase (choline to phosphocholine), phosphocholine cytidylyltransferase [phosphocholine to cytidine diphospho (CDP)-choline], and finally 1,2-diacylglycerol choline phosphotransferase (CDP-choline to PC). Cells infected with the wild-type SP showed elevated CDP-choline levels, indicating impairment of the final enzymatic step in PC synthesis. Supplementation with external lyso-phospatidylcholine prevented apoptosis in microglia and neurons along with inhibition of AIF release (Zweigner et al., 2004). Inhibition of PC synthesis was abrogated by use of pneumolysin and hydrogen peroxide production-mutants, implicating these virulence factors in this process. In addition, when citicholine (cytidine diphosphocholine) was administered 24 h prior to and at the time of SP infection, there was a significant reduction in apoptotic neurons in the hippocampus of mice, compared to those that received SP alone. Presumably, the excessive CDP-choline, when administered *in vivo* was able to “overcome the bacterial-induced partial inhibition of the final enzyme in the pathway” (Zweigner et al., 2004). The precise mechanism of the PC synthesis inhibition by SP virulence factors is unclear.

Thrombopoietin (Tpo) is a glycoprotein synthesized mainly in the liver and kidneys that is involved in regulation of platelet production. Other than in hematopoietic tissues, its expression has been demonstrated in the brain as well, and has been shown to have a pro-apoptotic effect on neurons by activation of a caspase-dependent pathway. In primary hippocampal neurons, externally administered Tpo was shown to activate ERK, and caspase-3 activation was seen in the cortex regions of rats receiving Tpo (Ehrenreich et al., 2005). *In vitro* inhibition of either ERK or caspase-3 completely downregulated Tpo-induced neuronal apoptosis. In a recent study, in a mouse model of SP meningitis, *Tpo* mRNA was shown to be upregulated at 12 h and 24 h post-infection (Hoffmann et al., 2011), and mice lacking the Tpo receptor (*c* − *Mpl*^−/−^) showed reduced microglial activation as determined by the CD45 marker. Most importantly, these knock-out mice also had reduced neuronal apoptosis in the dentate gyrus region. In addition, while the wild-type conditioned medium was able to upregulate *Tpo* mRNA from primary microglia, conditioned medium from a pneumolysin or hydrogen peroxide-defective mutants did not, again implicating a complex and multifactorial role for these virulence factors in neuronal apoptosis.

Interestingly, the excitatory amino acid glutamate can also induce neuronal apoptosis by mechanisms that are similar to those induced by live pneumococci, pneumolysin, and hydrogen peroxide. Glutamate toxicity has been shown to induce calcium influx in neuronal cells (Keelan et al., 1999), followed by activation of calcium-dependent proteases like calpain leading to release of AIF from mitochondria (Zhang and Bhavnani, 2006). In some neuronal cells these changes are accompanied by caspase-3 activation as well. Glutamate levels in the CSF have been shown to be upregulated in bacterial meningitis due to SP as well as other bacteria (Guerra-Romero et al., 1993; Ma et al., 2003). However, despite the mechanistic similarity with pneumococci in inducing neuronal apoptosis and its elevated levels in CSF, the role of this neurotransmitter in SP meningitis is not clear. In one study, measurement of the hippocampal glutamine synthetase (GS) activity, (the enzyme that converts toxic glutamate to glutamine), showed no change following meningitis in rabbits, while the cortical GS activity was significantly upregulated (Tumani et al., 2000). When animals were injected with a GS inhibitor post-infection, neuronal apoptosis was elevated significantly in the hippocampus region. Presumably, during natural infection, lack of elevated levels of GS in the hippocampus would lead to an inability to detoxify the excess glutamate levels, resulting in apoptosis. In contrast, inhibition of a selective glutamate receptor NR2B, reduced seizures in infant rats, but had no effect on dentate gyrus neuronal apoptosis (Kolarova et al., 2003). It is possible that other types of glutamate receptors are involved.

The roles of reactive oxygen and nitrogen species in inducing hippocampal cell apoptosis in SP infection have also been evaluated, with mixed results for both. Antioxidant N-acetyl-L-cysteine (NAC) when used in a rat model of SP meningitis had a protective effect and alleviated intracranial pressure (ICP), reduced CSF leukocyte count and the water content in the brain. However, its effect on neural cell apoptosis was not reported (Koedel and Pfister, 1997). In another study, NAC and other clinically used antioxidants were shown to reduce necrotic cortical injury in infant rats but not apoptosis in the hippocampal region (Auer et al., 2000). There was no significant change in CSF bacterial titer or TNF levels either. In a third study, the free radical scavenger PBN (α-phenyl-*tert*-butyl nitrone) was shown to actually exacerbate hippocampal apoptosis during SP-mediated meningitis in infant rats (Loeffler et al., 2001). In contrast, iNOS-deficient mice exhibited reduced neuronal loss (Hoffmann et al., 2006) and caspase-3 activation in the brain (Braun, 2009). They also produced significantly lower levels of IL-6, IL-1β, and TNF proteins as well as lower levels of CCL3 and CXCL2 mRNA in the brain following SP infection (Winkler et al., 2001). However, use of S-methylisothiourea, an iNOS inhibitor, had no effect on the pathophysiology as measured by ICP, CSF white blood cell count, etc., in a rat model of SP meningitis, although apoptosis was not measured (Koedel and Pfister, 1997). The discrepancies are likely due to differences in animal models, pharmacokinetic parameters and nonspecific, random effects (Braun, 2009).

### Group B *streptococcus* (GBS)

GBS, a gram-positive bacterium, is a preeminent agent of neonatal sepsis in Western countries, and the third leading cause of meningitis due to bacteria in the US (Schuchat et al., 1990; Schuchat, 1999) (Table 1). Nearly 50% of meningitis cases in newborn patients can be attributed to GBS alone (Lehnardt et al., 2006). As with SP, GBS infection in infants results in injury to brain cells, leading to long-term neurological sequelae. In an infant rat model of GBS meningitis, intracisternal injection of the bacteria led to the development of both apoptosis in the hippocampus, and necrosis in the cortex, along with the production of reactive oxygen intermediates (ROI) (Leib et al., 1996a). Administration of a free-radical scavenger at the time of infection led to complete elimination of both forms of injury, while a post-infection treatment with the scavenger mitigated apoptosis much more so than necrosis, although both mechanisms of cell death were dampened. In a subsequent study by the same group, administration of a TNF antibody also led to decrease in neuronal apoptosis but not cortical necrosis (Bogdan et al., 1997). TNF can trigger the extrinsic pathway of apoptosis, and also induce ROI production. ROI in turn can activate apoptosis signal-regulating kinase 1 (ASK1) and transcription of pro-apoptotic genes through the AP1 transcription factor and/or the intrinsic pathway through mitochondrial disruption and release of cytochrome c (Circu and Aw, 2010). TNF, however, could also modulate activation of excitatory amino acid receptors, which in turn can induce apoptosis of neurons (Gelbard et al., 1993; Zhang and Zhu, 2011). Administration of kynurenic acid, an antagonist of excitatory amino acids, also had a protective effect on both hippocampal and cortical neurons during GBS meningitis (Leib et al., 1996b). Although the contribution of these individual factors has been determined to play a role in neuronal apoptosis, the exact pathways have not been delineated as yet.

While infection with GBS induces necrosis in the cortex, exposure of primary cortical neurons to heat-killed (HK) GBS has been shown to induce apoptosis. Both HK-GBS and a secreted protein factor from GBS were able to induce neuronal apoptosis, but only in the presence of microglia. Moreover, HK-GBS can also induce microglial apoptosis. These phenomena are time-dependent, with the neuronal apoptosis occurring earlier at 18 h and the microglial death after 32 h of incubation with HK bacteria. In both microglia and neurons the TLR2/MyD88 pathway was shown to play a significant role in inducing apoptosis, as microglia derived from *Tlr2*^−/−^ or *Myd88*^−/−^ mice do not die themselves or cause neuronal death in the presence of HK-GBS. Interestingly, apoptosis of neurons in the presence of microglia by HK-GBS is nitric-oxide (NO)-dependent, as inhibition of microglial NO production by aminoguanidine abrogated neuronal apoptosis, while it had no effect on microglial apoptosis. Microglial apoptosis via the TLR2/MyD88 pathway activates caspase-8, but not caspase-3 (Lehnardt et al., 2006, 2007). Whether a similar pattern of caspase activation is seen in cortical neurons is not known.

As for the role of bacterial factors in inducing apoptosis, GBS LTA was shown not to be involved in either the induction of NO from microglia or in microglial apoptosis. However, recently, the GBS toxin beta-hemolysin was shown to play a role in neuronal apoptosis that was caspase-independent (Reiss et al., 2011). Additionally, contrary to previously published results (Leib et al., 1996a; Bogdan et al., 1997), in this study live GBS infection was able to induce neuronal apoptosis in the cortex as well. Whether these differences are attributable to different strains of rat (Sprague Dawley vs. Wistar), bacterial inoculum size and/or experimental procedures, remains to be determined (Reiss et al., 2011).

## Other bacteria

The following section summarizes the apoptotic mechanisms induced in the CNS by rare/uncommon infections and those bacterial infections that are frequent but for which limited data on CNS apoptosis is available (Table 1).

### Brucella abortus

*B. abortus* is a gram-negative, facultative intracellular bacterium that causes disease both in cattle and in humans (Christopher et al., 2010). Human neurobrucellosis, a rare but morbid condition, manifests in many forms, including meningitis, encephalitis, brain abscesses, myelitis, strokes, and psychiatric disorders (Garcia Samartino et al., 2010; Obiako et al., 2010). It is considered an infectious disease of inflammatory pathogenesis that is associated with microglial activation and astrogliosis. In an experimental murine model of neurobrucellosis, intracranial inoculation with HK *B. abortus* led to proliferation of astrocytes with concurrent apoptosis, which characterize astrogliosis. Also, several pro-inflammatory mediators including IL-6, IL-1β, TNF, CCL2, and CXCL1 were shown to be released from primary glial cell cultures (microglia and astrocytes) in response to the non-viable bacteria. These effects were mimicked by lipidated Omp19, a lipoprotein, but not by its unlipidated form, implying a role for the lipid portion in inducing inflammation and astrocyte apoptosis. No effect of *B. abortus* LTA was seen on chemokine or cytokine production from either astrocytes or microglia. In addition, TNF receptor-1 knock-out mice (*Tnfrp55*^−/−^) or caspase inhibition prevented astrocyte apoptosis, indicating a role for the extrinsic pathway of apoptosis in astrocyte cell death (Garcia Samartino et al., 2010).

### Listeria monocytogenes

*L. monocytogenes* is a gram-positive, facultative intracellular, food-borne pathogen that causes gastroenteritis, sepsis, and meningitis. As with *B. abortus*, infection with this bacterium can cause abortions and has a significant mortality rate in immunocompromised individuals (Gray and Killinger, 1966; Sleator et al., 2009). *L. monocytogenes* can induce cell death in diverse cell types, by different means. In mice, death of hepatocytes, B cells, and dendritic cells was shown to be induced by apoptosis while macrophages and human amniotic cells died by necrosis (Ammendolia et al., 2009) in response to *Listeria* infection. Although post-mortem analysis of patients with listeriosis has shown mostly necrotic lesions and other pathological signs in the brain (Drevets et al., 2004), experimental infection of mouse CNS with *Listeria* has also been shown to induce apoptosis in cerebellar and hippocampal neurons (Schluter et al., 1998), as well as in a mouse neuronal cell line Neuro-2a *in vitro* (Parra et al., 2008). In the latter study a prominent and dose-dependent role for the cytolytic listeriolysin O (LLO) in inducing cell death was established. The exact mechanism of LLO-mediated cell death is not known although hypotheses such as Ca^2+^ influx leading to mitochondrial dysfunction and/or a dose-dependent activation of the MEK pathway, resulting in apoptosis were put forth (Parra et al., 2008).

### Staphylococcus aureus

*S. aureus*, a gram positive, facultative anaerobic bacterium is one of the most common causative agent of brain abscess. Experiments in animal models have indicated that during abscess formation, activation of microglia, astrocytes, and neutrophils is common, along with edema and tissue necrosis (Kielian et al., 2007; Esen et al., 2010). Meningitis, caused by hematogenous spread, or acquired post-neurosurgery is of rising incidence and importance especially due to an increase in methicillin-resistant *S. aureus* (MRSA) strains in recent years. Although MRSA-mediated meningitis is rare, it has a high mortality rate, and is therefore a very serious disease (Aguilar et al., 2010; Pintado et al., 2012). Studies on the effect of *S. aureus* LTA on rat brain cells have shown that it can induce cerebellar neuronal cell death but only in the presence of glial cells. Inhibition of NO had a moderate effect in preventing cell death, while superoxide and peroxynitrite inhibition had a more profound effect on neuronal viability. LTA-induced neuronal cell death occurred both by necrosis and apoptosis, and inhibition of caspase-3 or 8 significantly improved cell viability, but did not completely eliminate cell death. Although glial cells produce an inflammatory milieu by the secretion of TNF, IL-1β, and IL-6 upon exposure to *S. aureus* LTA, no effect of TNF or IL-1Rα antibody was seen in terms of neuronal cell viability (Kinsner et al., 2005). In subsequent studies it was shown that the LTA-induced production of TNF, IL-6, IL-1β, and NO from primary microglia was through TLR2 and downstream p38 and ERK1/2 pathways and that muramyl dipeptide, a component of peptidoglycan, can potentiate NO production (Kinsner et al., 2006).

### Borrelia burgdorferi

Lyme borreliosis, caused by the gram-negative spirochete *B. burgdorferi*, is a vector borne disease transmitted through the bite of infected ticks. The neurological manifestations of Lyme disease, termed Lyme neuroborreliosis (LNB), occur in about 15% of patients with erythema migrans, the typical bull's eye-shaped skin rash that develops at the site of the tick bite. LNB affects both the CNS and PNS, resulting in numerous signs and symptoms, including meningitis, meningoencephalitis, radiculitis, and several neuropathies. Experimental infections of rhesus macaques with *B. burgdorferi* indicate that, as with many other CNS infections, inflammation occurs in response to the presence of *B. burgdorferi* in the CNS, accompanied by neuronal and glial loss. *In vitro* experiments point to an apoptotic form of cell death in neurons and oligodendrocytes (Ramesh et al., 2003, 2012; Myers et al., 2009). In a manner similar to other bacterial CNS infections, neuronal loss occurs in the presence of microglia, which in turn is the generators of an inflammatory milieu composed of many chemokines and cytokines including CCL2, TNF, IL-6, IL-8, and others (Myers et al., 2009). However, addition of exogenous TNF, IL-6, and IL-8 had no effect on neuronal apoptosis, indicating that other factors are involved in this process. Interestingly, a microarray analysis of neuronal gene transcription in the presence of microglia and *B. burgdorferi* showed a role for p53 regulated genes. A role for microglial TLRs was also implied with the up-regulation of TLR2 mRNA in response to *B. burgdorferi*, as well as protein staining of TLRs 2, 4, and 5 and a pro-inflammatory response by microglia to TLR2 and TLR5 agonists (Bernardino et al., 2008). Thus, it is likely that TLR-mediated events in microglia control inflammatory mediator release, which in turn could induce apoptosis in neurons, perhaps through a p53-related pathway. Oligodendrocytes, on the other hand, undergo apoptosis in the presence of *B. burgdorferi* alone (Ramesh et al., 2012). Similar to microglia, an inflammatory environment composed of CCL2, IL-6, and IL-8 has been demonstrated. Down-regulation of oligodendrocyte inflammation *in vitro* with the steroid dexamethasone also diminished oligodendrocyte apoptosis, indicating a role for inflammatory pathways in glial cell apoptosis. The precise mechanisms under which these cells undergo apoptosis are not currently known. In rhesus macaques, infection with *B. burgdorferi* has been shown to induce astrogliosis as well, wherein astrocytes proliferate and undergo apoptosis (Ramesh et al., 2003).

### Chlamydophila (chlamydia) pneumoniae

*C. pneumoniae* is an obligate intracellular gram-negative bacterium that has been associated with a number of diseases ranging from atypical pneumonia, sinusitis, meningoencephalitis, and more recently Alzheimer's disease (Grayston et al., 1993; Balin et al., 1998; Guglielminotti et al., 2000; Blasi et al., 2009). It is a traditionally respiratory pathogen than can spread to far-off tissues (Boelen et al., 2007b). *C. pneumoniae*, unlike most CNS pathogens, has been shown to predominantly inhibit apoptosis of host cells, including epithelial cells, neutrophils, THP-1 monocytes as well as microglial cells (Fan et al., 1998; Fischer et al., 2001; Rajalingam et al., 2001; Airenne et al., 2002; Van Zandbergen et al., 2004; Boelen et al., 2007b; Appelt et al., 2008), although data do exist that show that this organism may also induce apoptosis (Goth and Stephens, 2001; Sessa et al., 2009; Olivares-Zavaleta et al., 2011). Suppression of apoptosis was due to a combination of many factors, including down-regulation of cytochrome c release and caspase-3 activation, destruction of the pro-apoptotic Bcl-2 family of proteins (Bim, Puma, and Bad in particular) (Fischer et al., 2004), and through the anti-apoptotic IAPs (Paland et al., 2006). While live bacteria were able to induce this anti-apoptotic phenotype, HK bacteria were unable to induce apoptotic resistance in some monocytic cells indicating a role for a bacterial heat—labile factor in this phenomenon (Carratelli et al., 2002). With regard to neuronal cells, infection with *C. pneumoniae* has been shown to induce varied phenotypes in terms of cell death. In the human neuroblastoma cell line SK-N-MC, *C. pneumoniae* infection was shown to down-regulate apoptosis by reducing caspase-3/7 activity even in the presence of the classical apoptosis inducer staurosporine, and establish a persisting infection (Appelt et al., 2008). In contrast, a murine neuronal cell line NB41A3 (and to some extent murine astrocytes) were highly susceptible to cell death and underwent necrosis when exposed to *C. pneumoniae in vitro* (Boelen et al., 2007b). In addition, when conditioned, filtered medium from a murine microglial cell line previously infected with the bacteria was added to NB41A3, the latter cells were shown to undergo significant apoptosis as well as necrosis (Boelen et al., 2007a). Cell death was primarily mediated through TNF and IL-6, as antibodies to these cytokines in the conditioned medium reduced neuronal cell death (due to both apoptosis and necrosis) by half.

### Neisseria meningitidis

This gram-negative organism, also known as meningococcus, is another prominent cause of meningitis in the post-natal period (Kim, 2003). *N. meningitidis* like *Chlamydophila*, mostly suppresses apoptosis in a variety of cell types, including macrophages, umbilical vein endothelial cells, and HeLa (epithelial) cells (Massari et al., 2003, 2010; Linhartova et al., 2006; Tunbridge et al., 2006). Adhesion molecules that suppress pro-apoptotic gene expression, meningococcal porin (PorB) that stabilizes mitochondrial membrane, and bacterial enzymes that mediate nitric oxide detoxification are some of the predominant factors involved in this anti-apoptotic process. In some cases, pro- or anti-apoptosis seemed to depend on the pathogenicity of the infecting strain. In human epithelial cells, asymptomatic carriage strains provoke the shedding of TNFR1 and prevent apoptosis, while the pathogenic strains induce TNF production and subsequent apoptosis (Deghmane et al., 2009). In other cases, it seemed to be host-species or tissue-dependent, or a function of the type/nature of the stimuli. In mouse cerebrovascular endothelial cells, sonicated lysates of meningococcus induce cell death through NO, as NO inhibitors down-regulate cell death (Constantin et al., 2002). However, these cells do not display any signs of apoptosis, such as caspase-3 activation, DNA fragmentation, or phosphatidyl serine translocation. Conversely, in human BMECs, caspase-3 activation, phosphatidyl serine translocation, and apoptotic gene transcription in response to live *N. meningitidis* has been reported (Schubert-Unkmeir et al., 2007).

### *Escherichia coli* K1

The gram-negative *E. coli* carrying the capsular antigen K1 is another leading cause of neonatal meningitis, like GBS (Kim, 2003). However, unlike GBS, which has been shown to induce apoptosis in a variety of cell types, including CNS cells as well as macrophages, epithelial cells, and other non-CNS tissues (Fettucciari et al., 2000; Beyrich et al., 2011; Da Costa et al., 2011; Oliveira et al., 2012) *E. coli* K1 has been shown to inhibit apoptosis in such cells (Sukumaran et al., 2004, 2010). Inhibition of apoptosis in both macrophages and epithelial cells was through down-regulation of cytochrome c release. In macrophages this was through bacterial OmpA-mediated upregulation of the anti-apoptotic Bcl-xL, while in epithelial cells it was through the stabilization of mitochondrial voltage-dependent anion channel (VDAC)—hexokinase (HK) association by bacterial FimA. HK has been shown to dissociate from VDAC prior to cytochrome c release in response to apoptotic signals (Pastorino et al., 2005). By strengthening the association between VDAC and HK, dissociation of HK from mitochondria was prevented thereby inhibiting cytochrome c release.

With regard to meningitis and CNS, a preponderance of laboratory work with this bacterium has been focused on the blood-brain barrier, and not on neuronal cells. It was recently shown that the Hcp1 protein, which is secreted from the bacterium through a putative Type VI secretion machinery, was able to induce BMEC apoptosis by activation of caspase-8 but not caspase-3; this occurred together with the production of IL-6 and IL-8 (Zhou et al., 2012). It was hypothesized that Hcp1 directly binds a receptor, thereby activating caspase-8. However, no TNF or IL-1β was measured, or experiments performed to determine the role, if any, of these molecules in the induction of apoptosis.

The effect of *E. coli* K1 meningitis on neuronal apoptosis has been documented in a few laboratory investigations. In one study it was shown that intra-cisternal injection of *E. coli* K1 in rabbits resulted in neuronal apoptosis in the hippocampus region (Spreer et al., 2006), and administration of dexamethasone aggravated this condition. In another study, it was shown that while *E. coli* was similarly able to induce hippocampal neuron apoptosis via intranasal administration in newborn mice, brain pathology was eliminated in iNOS-deficient mice as well as in mice receiving NO inhibitors (Mittal et al., 2010). This was predominantly due to the clearance of bacteria in the blood and reduced permeability of the blood-brain barrier in NO-deficient animals. Since dexamethasone has been shown to down-regulate iNOS mRNA induced in rat brain in response to LPS (Suzuki et al., 1997; Wang et al., 2005), the discrepancies between those two studies are not completely clear. Since NO levels were not measured in the former study (Spreer et al., 2006), a direct comparison is not possible. Whether the differences in animal models and route of bacterial administration account for altered apoptotic phenotypes remains to be determined.

### Klebsiella pneumoniae

*K. pneumoniae* is a gram-negative bacterium that is an emerging cause of CNS infections (Fang et al., 2007). *In vivo* experiments in rats have demonstrated the neurodegenerative effects of *K. pneumoniae* infection in the brain, resulting in apoptosis of neurons in the hippocampus as well as the cortex in some cases (Chiu et al., 2011; Wu et al., 2011). Bacterial meningitis induced by *K. pneumoniae* was shown to up-regulate IL-6, IL-1β, and TNF production in the CSF, predominantly by microglia and astrocytes (Wen et al., 2007). Control of inflammation by melatonin administration resulted not only in down regulation of these cytokines, but also engendered neuroprotection (Wu et al., 2011). Thus, the pro-inflammatory cytokines (and likely reactive radicals) are involved in *K. pneumoniae*-mediated neuronal apoptosis.

### Rickettsia rickettsii

*R. rickettsii* is an obligate intracellular gram-negative bacterium and is the causative agent of tick-borne Rocky Mountain spotted fever. CNS involvement occurs in the later stages of the disease, with reported manifestations ranging from meningoencephalitis, seizures, blindness, deafness, and paralysis, to coma and death (Bleck, 1999; Joshi and Kovacs, 2007). In one study, the organism was shown to induce apoptosis of cerebellar granule neurons *in vitro* (Joshi and Kovacs, 2007). However, no mechanism is currently known.

## Concluding remarks

Neural cell apoptosis caused by bacteria in the CNS may be associated with severe neurological sequelae and long-term disabilities—particularly since neuronal regeneration is incomplete and glial cell recuperation may be prolonged. Therefore, it is imperative to elucidate mechanisms of apoptosis in specific infections, to design better therapeutic interventions. As this review highlights, this is no simple task, since apoptotic pathways vary widely among tissues and cell types, which in turn respond differently to whole bacteria or their virulence factors, and to the inflammation these induce. In addition, as even dead or lysed bacteria can induce inflammation and/or cell death in some cases, additional therapy by means of anti-inflammatory mediators should be considered. Finally, as the antibiotic repertoire is limited and antibiotic resistance continues to spread, it is possible that the incidence of CNS infections will rise. The development of novel therapeutics to combat such conditions needs to be addressed.

### Conflict of interest statement

The authors declare that the research was conducted in the absence of any commercial or financial relationships that could be construed as a potential conflict of interest.

## Footnotes

^1^Inhibition of caspase 8 on the other hand, after TNFR1 engagement, can lead to necroptosis by caspase-independent mechanisms.
